# Discover overlooked complications after preeclampsia using electronic health records

**DOI:** 10.21203/rs.3.rs-3937688/v1

**Published:** 2024-03-06

**Authors:** Lana Garmire, Haoming Zhu, Xiaotong Yangs, Wanling Xie, Elizabeth Langen, Ruowang Li

**Affiliations:** University of Michigan; University of Michigan; University of Michigan; Cedars-Sinai Medical Center; University of Michigan; University of Pennsylvania

## Abstract

**Background:**

Preeclampsia (PE) is a severe pregnancy complication characterized by hypertension and end-organ damage such as proteinuria. PE poses a significant threat to women’s long-term health, including an increased risk of cardiovascular and renal diseases. Most previous studies have been hypothesis-based, potentially overlooking certain significant complications. This study conducts a comprehensive, non-hypothesis-based analysis of PE-complicated diagnoses after pregnancies using multiple large-scale electronic health records (EHR) datasets.

**Method:**

From the University of Michigan (UM) Healthcare System, we collected 4,348 PE patients for the cases and 27,377 patients with pregnancies not complicated by PE or related conditions for the controls. We first conducted a non-hypothesis-based analysis to identify any long-term adverse health conditions associated with PE using logistic regression with adjustments to demographics, social history, and medical history. We confirmed the identified complications with *UK Biobank* data which contain 443 PE cases and 14,870 non-PE controls. We then conducted a survival analysis on complications that exhibited significance in more than 5 consecutive years post-PE. We further examined the potential racial disparities of identified complications between Caucasian and African American patients.

**Findings:**

Uncomplicated hypertension, complicated diabetes, congestive heart failure, renal failure, and obesity exhibited significantly increased risks whereas hypothyroidism showed decreased risks, in 5 consecutive years after PE in the UM discovery data. *UK Biobank* data confirmed the increased risks of uncomplicated hypertension, complicated diabetes, congestive heart failure, renal failure, and obesity. Further survival analysis using UM data indicated significantly increased risks in uncomplicated hypertension, complicated diabetes, congestive heart failure, renal failure, and obesity, and significantly decreased risks in hypothyroidism. There exist racial differences in the risks of developing hypertension and hypothyroidism after PE. PE protects against hypothyroidism in African American postpartum women but not Cacausians; it also increases the risks of uncomplicated hypertension but less severely in African American postpartum women as compared to Cacausians.

**Interpretation:**

This study addresses the lack of a comprehensive examination of PE’s long-term effects utilizing large-scale EHR and advanced statistical methods. Our findings underscore the need for long-term monitoring and interventions for women with a history of PE, emphasizing the importance of personalized postpartum care. Notably, the racial disparities observed in the impact of PE on hypertension and hypothyroidism highlight the necessity of tailored aftercare based on race.

## Introduction

Preeclampsia (PE) is a severe pregnancy complication that emerges after the 20th week of gestation and is characterized by hypertension and end-organ damage [1]. Its prevalence in the United States is estimated at approximately 4%, with a global prevalence of 2–8% [3, 4]. PE profoundly impacts maternal and neonatal health, primarily through a hypertensive state that increases vascular resistance, potentially impairing blood flow to essential organs [2]. This condition leads to endothelial dysfunction, which can cause extensive damage to the mother’s kidneys, liver, and central nervous system [2]. Additionally, PE adversely impacts placental perfusion, potentially resulting in fetal growth restrictions and preterm births [2]. While the resolution of pregnancy often mitigates the acute symptoms of PE, its long-term effects on maternal health, manifesting as various chronic complications such as hypertension, cerebrovascular disease, diabetes, cardiovascular disease, and renal disease, can persist for years postpartum [8].

Despite the well-documented immediate consequences, there is a notable gap in a comprehensive examination of PE’s long-term impacts. Previous studies, including cohort studies and meta-analyses, have indicated the increasing risks of chronic hypertension, cardiovascular, renal, and metabolic disorders post-PE [5–9, 11–13, 21–26]. However, these studies adopted a hypothesis-based approach, focusing on confirming known associations and potentially overlooking important associations. Moreover, the majority of these studies utilized basic statistical models (e.g., t-tests, chi-square tests, linear regression) [5–8, 11–13, 21–26] despite the need for more robust analytical methods to capture crucial time-to-event information and other competing factors (e.g. death, loss of followup), especially when dealing with longitudinal data over a long period. Moreover, most of these studies did not adjust for significant confounders, especially for pre-existing conditions, or only adjusted for basic confounders such as age, race, and gestational age [5–7, 11, 13, 21–24]. This left the causal relationship of PE and researched complications unclear. In addition, few studies have paid attention to the racial disparities in PE’s long-term effects. As statistical methods evolve, a more robust and comprehensive approach utilizing modern analytical methods and large datasets is imperative to explore the long-term effects of PE and offer clinical insights.

With the widespread use of electronic medical systems, the Electronic Health Record (EHR) has become an important source of patient data for medical research. EHR provides comprehensive and timestamped patient information in large volumes, including demographics, diagnosis records, social history, laboratory results, etc. These features make it compatible with modeling quantitative outcomes, such as patient survival. Survival analysis enables the examination of time until the occurrence of specific events [10]. Compared with basic statistical models such as t-tests and linear regression, survival analysis offers advantages in terms of handling censorship, accommodating non-normally distributed data, and providing a more detailed understanding of disease trajectories. Incorporating survival models into the analysis offers a better understanding of PE’s long-term consequences.

This study adopts a non-hypothesis-based exploration, leveraging EHR data to investigate connections between PE and subsequent health trajectories. Our goal is to first identify any long-term complications significantly associated with PE and then comprehensively study their trajectories, as well as potential racial disparities. Through such analysis, we aim to enhance the clinical understanding of PE’s long-term consequences and ultimately help care providers adopt preventive aftercare strategies.

## Methods

### Data Source

We obtained original EHR data for two patient cohorts: the discovery cohort and the confirmation cohort. The discovery data are from the *University of Michigan (UM) Medicine Healthcare System*, an academic health system in Michigan State. The University of Michigan Medical School’s Institutional Review Board (IRB) granted data utilization approval under HUM#00168171. The EHR from the *UM Medicine Healthcare System* provides comprehensive features, including diagnoses, encounter information, demographics, medications, etc. The complete records for these features are available starting in 2006 [38]. We obtained confirmation data from the *UK Biobank*, a long-term study with approximately 500,000 volunteers, providing similar features [31]. All *UK Biobank data* for the confirmation cohort pertained to project 86494. These records are available starting in 2006. Our study initially utilized features including diseases diagnosed, age at diagnosis, medical history, pre-existing complications, race, and social history.

The discovery data comprise case patients having at least one PE diagnosis between 2003 and 2023, and the controls having at least one pregnancy during the same period but no diagnosis of PE-related diseases (PE, eclampsia, pre-existing hypertension complicating pregnancy, gestational edema, and maternal hypertension). Inclusion and exclusion criteria were based on the International Classification of Diseases (ICD)-9 and − 10 codes [14, 15]. We followed the same selection criteria for the confirmation data from the *UK Biobank*. Detailed case-control selection criteria based on ICD-9 and ICD-10 codes are listed in **Supplementary Table 1**.

### EHR feature engineering

The diagnoses in EHR were initially recorded using a combination of ICD-9 and ICD-10 codes. We first standardized all diagnoses to ICD-10 using the “touch” package [28] to ensure consistency. Subsequently, we classified diagnoses into 31 medical complications, such as uncomplicated hypertension, complicated diabetes, obesity, etc., according to the Elixhauser Comorbidity Index [16]. The Elixhauser comorbidity index is a widely used method in healthcare to comprehensively assess and quantify the severity of multiple health conditions a patient might have [17]. A complete list of matches between ICD-10 codes and Elixhauser Comorbidities is shown in **Supplementary Table 2**.

To investigate the disease risk change after PE, we calculated the cumulative risk of each disease at intervals of one year, two years, and up to ten years after PE/normal pregnancy. We measured the follow-up length of each patient, defined as the time interval between their most recent record and their first PE diagnoses (for cases) or normal pregnancy (for controls). Then, for each disease, we calculated its risk in N years after PE, by comparing the occurrence of disease within N years in patients with at least N year follow-up length (N = 1, 2, …10).

Pre-existing complications of PE are confounders to be adjusted for in this study. For each patient, pre-existing complications are defined as those presented at or before their first PE (for cases) or normal pregnancy (for controls) diagnosis. We encoded the pre-existing complications as binary entries (1 for presence, and 0 for absence) following the Elixhauser Comorbidity Index, as well as additional features, including PE history, gestational diabetes history, and substance use (alcohol and smoking status). To enhance statistical power and reduce model complexity, we removed features with more than 20% missing values or a p-value > 0.10 in the initial univariable test as advised [41]. We applied the feature selection process independently to each group. The complete list of final features in each group is shown in **Supplementary Table 3**.

### Non-hypothesis-based PE-induced complications discovery

In each group (N = 1, 2, … 10), we applied logistic regression to the complications that reached the effective sample size recommended for clinical logistic regression [18, 39]. We then introduced a binary variable to indicate the presence of complications after the first PE (for cases) or normal pregnancy (for controls) diagnosis of each patient, as the response variable in logistic regression. A value of 1 means the patients had the complication after the first PE (for cases) or normal pregnancy (for controls) diagnosis, and 0 otherwise.

Using the R package “glmnet” [18], we fitted a logistic regression model for each complication in each group, and recorded the regression coefficient, standard error, and p-value for predictor “PE”. We calculate the odds ratios (OR) and corresponding 95% confidence intervals (CI) based on the results. OR is a measure of the association between the presence of a particular condition and a specific outcome, expressing the odds of the event occurring in the cases relative to those in the controls [36]. We considered the complications that show significance in OR (p < 0.05) in more than 5 consecutive years to be significantly associated with PE. We further calculated Variance Inflation Factors (VIF) using the package “qacReg” [35] to check for potential collinearity among independent variables.

### Confirmation of complications after PE using UK Biobank data

To confirm the significant complications identified in the UM database, we constructed new logistic regressions on these complications in *UK Biobank* data following a similar process. A binary variable indicating the presence of complication after the patient’s first PE (for cases) or normal pregnancy (for controls) diagnosis serves as the response variable, while PE history, pre-existing complications, social history (smoking and alcohol-use status), and race serve as the regressors. We checked the OR and CI of the feature “PE” in each complication’s model for confirmation.

### Inspection of identified PE-induced complications and their racial disparities

We conducted survival analyses on the complications that are significantly associated with PE to further explore the disease trajectories. Patients who lost follow-up before the occurrence of events of interest were marked as censored. We built a Cox proportional-hazards (Cox-PH) survival model [19] for each significant complication. To further account for unrelated deaths’ effect on patient statistics, we adjusted the Cox-PH models for competing risks caused by unrelated deaths. The competing risks model was built using the “tidycmprsk” package [20]. Features used in the models can be found in **Supplementary Table 4**. We calculated the Hazard ratios (HR) of the factor “PE” in each model. HR is a measure used in survival analysis to compare the event rates at any given time point between two populations [37], which indicates the complication risks caused by PE in our study.

To examine potential racial disparities in PE’s effects, we further stratified patients according to their races (Caucasian, African American, Asian, American Indian or Alaska Native, Native Hawaiian and Other Pacific Islanders, Other Races, Unknown). We conducted comparative analysis on Caucasians (71.3%) and African Americans (13.8%), and omitted other races due to their very low representations in the population (less than 15% combined). We applied a similar survival analysis on complication risks and calculated the HR of PE on each complication for Caucasians and African Americans.

### Analytical tools

All analyses were conducted in R programming language v.4.0.3 [29], using packages “tidyverse” [33], “lubridate” [32], “glmnet” [18], “tidycmprsk” [20], “touch” [28], “survminer” [34], and “qacReg” [35].

### Data Sharing Agreement

The EHR used in this study contains sensitive patient information and cannot be made public. Researchers meeting the criteria for using sensitive data may contact the Research Scientific Facilitators at the University of Michigan Precision Health by emailing PHDataHelp@umich.edu or visiting https://research.medicine.umich.edu/our-units/data-office-clinical-translational-research/data-access for more information about requesting data access.

## Results

### Study overview and patient characteristics

**Figure 1:**
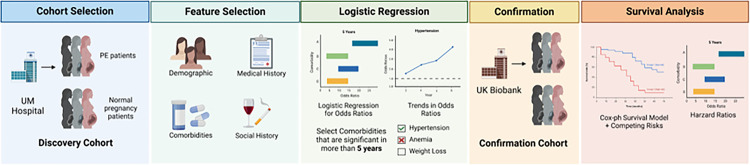
Project workflow. This figure shows the overall workflow. The Discovery Cohort was obtained from the *UM Healthcare System*, containing PE cases and non-PE controls. Feature selection was applied, followed by logistic regressions to identify complications after PE. The significant complications were further examined using survival analysis, and represented by hazard ratios. Patient data from the UK Biobank database were used to confirm the findings.

Using two large EHR datasets, we are the first to discover and validate the overlooked post-PE complications following a non-hypothesis-based approach and rigorous confounder adjustment. Utilizing EHR from the *UM Medicine Healthcare System*, we incorporated logistic regression and survival analysis to comprehensively investigate the long-term effects of PE, identifying persistent complications and racial disparities. We subsequently validated the results using EHR data from the *UK Biobank*. The overall workflow of the study is shown in Fig. 1.

The discovery EHR dataset from *UM Medicine Healthcare System* includes 4,348 patients for the cases and 27,377 patients for the controls. The confirmation data from *UK Biobank* include 443 patients for the cases and 14954 patients for the controls. The overall patient characteristics are summarized in Table 1. In both discovery and confirmation data, we observed significant differences between the cases and controls in demographic and clinical factors. To account for the differences, we included these factors in the regression models as confounders.

### Discovery and confirmation of six complications related to PE’s long-term effects

**Figure 2:**
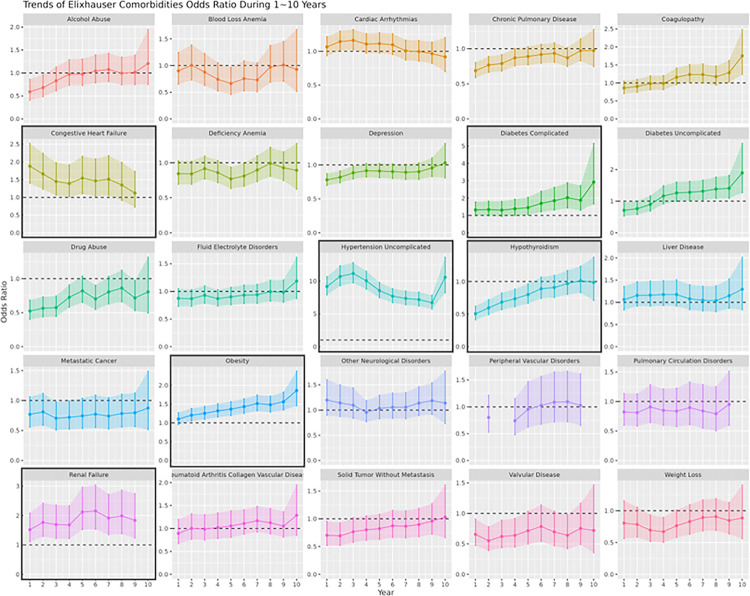
OR trends of all complications defined by Elixhauser Comorbidities. This figure shows the changes in OR of different complications within 1~10 years after a patient’s first PE (for cases) or normal pregnancy (for controls) diagnosis. For some years certain complications have inadequate effective size for analysis, leading to missing points in the plot. The dashed lines are OR = 1, where ORs above the line indicate higher risks of complications due to PE, and ORs below the line indicate lower risks of complications due to PE. Confidence intervals of the OR are indicated by the colored shadow areas. Plots of complications that are significant for at least 5 consecutive years are boxed.

We first investigated the change in all disease risks each year for 10 years after PE. We adopted the Elixhauser Comorbidity Index and categorized all ICD diagnosis codes into 31 Elixhauser disease categories (see **Method**). For each Elixhauser category, we applied logistic regression with PE as a predictor and whether or not diagnosed with the disease during N = 1 ~ 10 years as the response, adjusting for other factors such as age, pre-existing conditions, and social history. All features used in this study, such as complications, social history, and social history are listed in **Supplementary Table 3**. The population counts in each group (N = 1 ~ 10) are shown in **Supplementary Table 5**. If the population of a particular year falls below a reasonable effective size, we exclude that year from the plot to avoid bias(see Method). We then calculated the ORs of PE for each Elixhauser category and their CIs from these logistic regression models (Fig. 2). Some complications exhibit statistical significance in more than 5 consecutive years after the first PE (for cases) or normal pregnancy (for controls) diagnosis, during the 10-year period. These include uncomplicated hypertension (median OR = 8.85), renal failure (median OR = 1.84), complicated diabetes (median OR = 1.57), congestive heart failure (median OR = 1.47), obesity (median OR = 1.40), and hypothyroidism (median OR = 0.84). Notably, the ORs of PE for hypothyroidism are significantly lower than 1 in the first 5 years after patients’ first PE (for cases) or normal pregnancy (for controls) diagnosis, but with an overall elevating trend.

**Figure 3:**
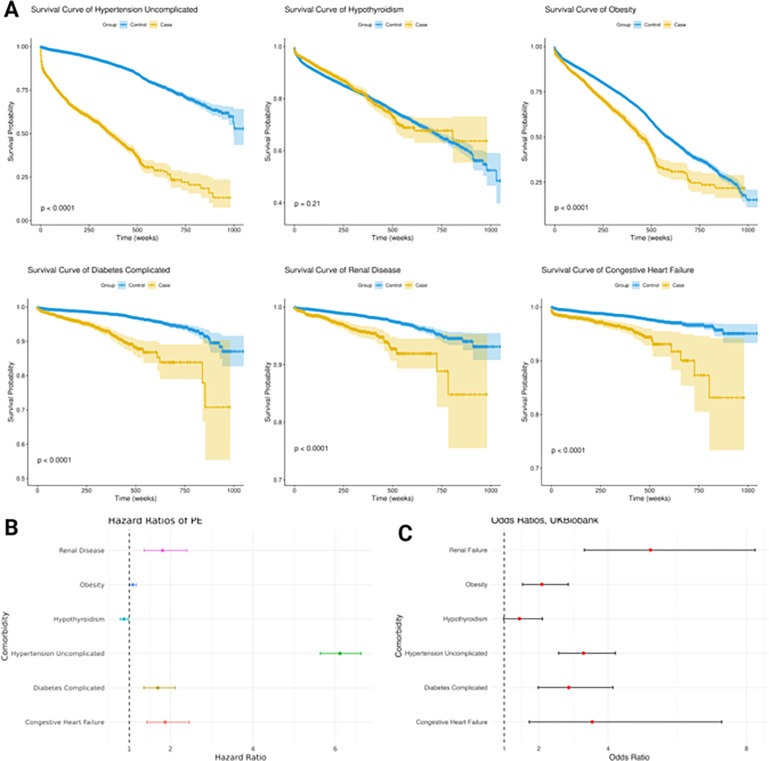
Characterization of significant complications after PE diagnosis. (A) Survival curves of significant complications of PE. The p-values are calculated from log-rank tests comparing the survival time of cases vs. controls. (B) Hazard ratios (HR) of Cox-PH survival models on the significant complications of PE, adjusted for competing risks. Data are represented as median HRs and error bars (the confidence intervals). (C) Confirmation of significant complications using *UK Biobank* Data. A new logistic regression is constructed using these complications and confounders. Data are represented as ORs from the logistical regression and error bars (the confidence intervals).

We then confirmed the significance of the six significant complications above, using an external dataset (UK Biobank) to ensure the generalizability of our discoveries. We refitted a logistic regression model over the same six complications on the *UK Biobank* data (Fig. 3C). Renal failure (OR = 5.22, 95% CI: 3.31–8.25), congestive heart failure (OR = 3.54, 95% CI: 1.72–7.29), uncomplicated hypertension (OR = 3.29, 95% CI: 2.57–4.21), complicated diabetes (OR = 2.87, 95% CI: 1.98–4.14), and obesity (OR = 2.09, 95% CI: 1.53–2.85) again show significant and increasing risks due to PE. Hypothyroidism (OR = 1.44, 95% CI: 0.99–2.11) is slightly below the significance threshold.

### Survival Analysis reveals the trajectories of identified complications after PE

We next conducted survival analyses of all six significant complications identified from the *UM Medicine Healthcare System*, to gain holistic views (Fig. 3A). All targeted complications show significant differences between the survival curves of the cases and controls (Log-Rank test, p < 0.05). For each of the six complications, we regressed the time until the first diagnosis of PE and confounders, such as age and pre-existing conditions, using the Cox-Proportional Hazard (Cox-PH) model. Diseases before developing the complication of interest were treated as competing risk events (see **Methods**). A complete list of the feature coefficients and significant levels is shown in **Supplementary Table 4**. We obtained the HRs and CIs specific to PE from the Cox-PH models and plotted them in Fig. 3B. Compared to women without a PE-related history, the HR of PE was 6.11 (95% CI: 5.64–6.63) for uncomplicated hypertension, 1.69 (95% CI: 1.35–2.12) for complicated diabetes, 1.87 (95% CI: 1.43–2.46) for congestive heart failure, 1.81 (95% CI: 1.36–2.40) for renal disease, 1.08 (95% CI: 1.00–1.17) for obesity, and 0.87 (95% CI: 0.78–0.97) for hypothyroidism.

### The racial disparities related to complications after PE

**Figure 4:**
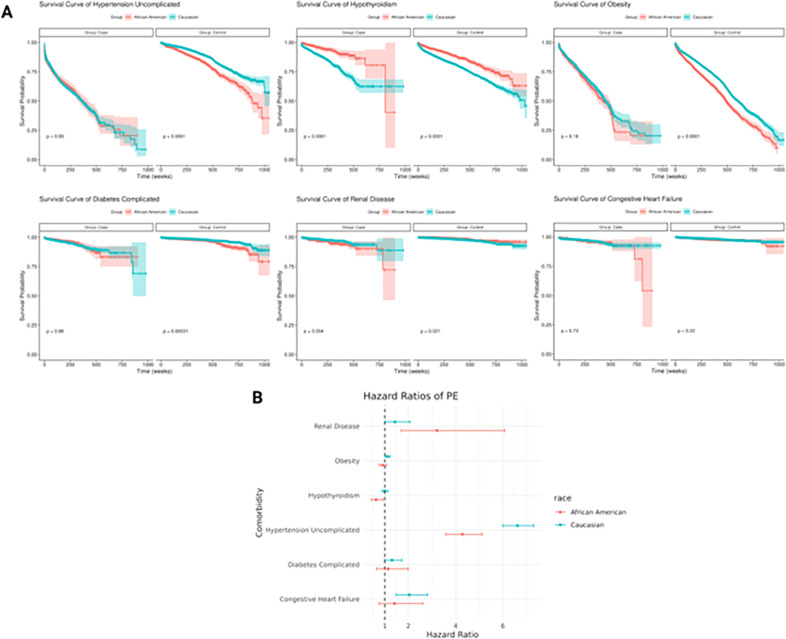
Racial disparities exhibited by complications after PE. (A) Survival curves of complications w.r.t. African Americans and Caucasians, separated by PE cases and controls. The p-values were calculated from log-rank tests comparing the survival time of Caucasians and African Americans in cases and controls, respectively. (B) Hazard ratios of complications due to PE w.r.t. African Americans and Caucasians. Hazard ratios are adjusted for competing risks.

Further, we stratified the patients according to their race and compared the difference between Caucasians and African Americans, who account for 71.3% and 13.8% of the UM cohort. We applied similar processes to the earlier but stratified on Caucasians and African Americans. The race-specific survival curves for each identified complication are shown in Fig. 4A. Hypothyroidism shows significant differences between the survival curves of African Americans and Caucasians within the cases (Log-Rank test, p < 0.05), where African Americans show better survival than Caucasians. We also applied Cox-PH models to each complication individually, to assess their associations with PE, while adjusting for confounders and competing risks. The race-specific HRs of PE are shown in Fig. 4B. After adjustment, we only observe significant racial disparities in hypothyroidism and uncomplicated hypertension. For hypothyroidism, PE’s effect on decreased hypothyroidism risk only occurs in African American postpartum women (HR = 0.63, 95% CI: 0.43–0.91), but is not significant in Caucasian postpartum women (HR = 1.00, 95% CI: 0.88–1.13). For uncomplicated hypertension, Cacausians postpartum women (HR = 6.62, 95% CI: 6.00–7.30) have significantly higher HR of PE (p < 0.05) than African American postpartum women (HR = 4.12, 95% CI: 3.50–4.85). Other complications also exhibit racial differences between African American and Caucasian postpartum women; however, the disparities are not significant.

## Discussion

This study employed a non-hypothesis-based approach and rigorous statistical analysis to comprehensively investigate and validate the overlooked long-term effects of PE for the first time. Using EHR from the *UM Medicine Healthcare System* and *UK Biobank* database, we discovered overlooked complications of PE and their racial disparities, in addition to confirming previously known conditions. These findings can encourage better management and interventions that benefit post-PE patient care.

The logistic regression models identified six complications from the *UM Medicine Healthcare* database—uncomplicated hypertension, complicated diabetes, congestive heart failure, renal failure, obesity, and hypothyroidism—that exhibited sustained significance over 5 or more consecutive years following PE, as indicated by their ORs. The increased risks of uncomplicated hypertension, obesity, complicated diabetes, renal failure, and congestive heart failure due to PE were confirmed using *UK Biobank* data. We then conducted survival analyses on all six complications, further taking time-to-event factors into account and confirming the enduring impact of PE. All six complications showed significant increasing risks due to PE in the longitudinal analysis. The findings underscore the need for long-term monitoring and interventions for patients who have a PE history.

This study on PE’s long-term complications is by far the first non-hypothesis-driven, comprehensive statistical analysis using multiple large-scale EHR datasets, with systematic adjustment for multiple confounding factors (demographics, social history, medical histories, etc.). While previous studies have recognized the increasing risks of hypertension [5, 8, 11, 12, 13], diabetes [8, 24, 25], congestive heart failure [5, 6, 8, 13, 23], renal failure [8, 21, 22], and obesity [8, 24, 25] after PE, most of them focused a specific complication. They did not necessarily consider sophisticated statistical modeling that handles confounders, leading to simplistic or even potentially biased results. One novel finding of our study is the discovery of the apparent protective effect of PE against subsequent hypothyroidism and the racial disparities in such effect. We observe an overall lowering risk of hypothyroidism due to PE. Interestingly, after stratifying patients into Caucasians and African Americans, we only observed PE’s protective effect on hypothyroidism among African American women but not Caucasian women. One previous review study suggested that Cacausian patients were generally referred for consultation for hypothyroidism at a younger age compared to African American patients and that non-Cacausian patients have a higher probability of being underdiagnosed [27]. Therefore, it remains to be confirmed whether the racial disparities in hypothyroidism after PE are primarily related to genetic disparities or bias in the timing of diagnosis between the two races.

Another novel finding of our study is the discovery of racial disparities in PE’s effect on subsequent hypertension. While the racial disparities in general hypertension have been widely recognized [46], there is a lack of research on disparities in PE’s inducing effect on subsequent hypertension. Among postpartum women without a PE history, we observe that African Americans have a significantly higher risk of hypertension than Caucasians, which is consistent with previous findings [46]. However, when taking into account PE’s effects and adjusting for confounders, we observe that Caucasians have significantly higher HR of PE on hypertension than African Americans. This potentially indicates that Caucasians are more sensitive to PE’s inducing effect on subsequent hypertension than African Americans.

Our study has several notable strengths compared to previous studies on the long-term effects of PE. Methodologically, we utilized a non-hypothesis-based approach to identify any association between complications and PE, allowing for a comprehensive investigation beyond hypothesis-driven approaches to allow the discovery of overlooked associations. We adjusted the analysis for pre-PE medical histories, minimizing the bias due to pre-existing conditions as many of them are indeed associated with PE. This addressed the pitfall in traditional case-control matching studies with only t-tests as statistical evidence [7, 9, 11–13]. In each step of our analysis, we conducted rigorous inference that ensured sufficient sample size and statistical power. We adopted a discovery-confirmation research strategy from two different datasets, strengthening our findings’ generalizability and reliability. There are few previous studies on the racial disparities of PE’s long-term adverse effects. Our study fills the research gap in this topic, revealing the association between race and PE’s long-term effects. Overall, we extended the PE research to a novel dimension, encouraging future work from a racial perspective and promoting precision aftercare of PE, which is important given the known impact of systemic racism on maternal health outcomes [40].

While our study has provided valuable insights into the long-term effects of PE, it is important to acknowledge the limitations. One aspect pertains to the reliance on ICD codes for case-control identification. ICD coding practices can vary between institutions, which may introduce biases and inaccuracies to the original data. Furthermore, the complexity of maternal health, encompassing various genetic, environmental, and lifestyle factors, makes it challenging to delineate direct causal relationships between PE and long-term complications. Future studies incorporating causal inference or randomized controlled trials may offer more insights into the causal pathways between PE and subsequent health outcomes. Given the increasing recognized subtypes of preeclampsia, which exhibit different pathological processes, diagnosis time, symptoms, time to deliveries, as well as outcome, it may be necessary to stratify PE by subtype if the patient size is sufficiently large in future studies [42–44]. Lastly, our findings are purely based on EHR data. Other types of data that perturb the molecular and pathological processes, such as genetics and genomics data [45], would be beneficial to deepen the mechanistic understanding of PE’s long-term effects.

## Figures and Tables

**Table 1:** Patient Characteristics for UM Data and UK Biobank Data.

Feature		UM Data			UK Biobank Data		
		Statistics		P-value	Statistics		P-value
		Case (n = 4348)	Control (n = 27,377)		Case (n = 443)	Control (n = 14,870)	
**Age, Years**		32.340	32.970	< 0.001	37.630	36.290	< 0.001
**Race**	African American	0.190	0.130	< 0.001	0.061	0.036	0.014
	Caucasian	0.708	0.714	0.435	0.867	0.900	0.033
	Other	0.102	0.156	< 0.001	0.072	0.064	0.581
**Social History**	Smoking	0.343	0.304	< 0.001	0.508	0.540	0.219
	Alcohol	0.557	0.535	< 0.001	0.927	0.947	0.101
**Pre-existing Disease**	Gestational Diabetes	0.067	0.055	0.002	0.015	0.004	0.006
	Other Complicated Diabetes	0.039	0.005	< 0.001	0.007	0.001	0.024
	Hypertension Uncomplicated	0.222	0.021	< 0.001	0.007	0.002	0.035
	Hypothyroidism	0.114	0.065	< 0.001	0.007	0.002	0.128
	Obesity	0.240	0.064	< 0.001	0.000	0.001	1.000
	Renal Failure	0.026	0.006	< 0.001	0.000	0.000	1.000
	Congestive Heart Failure	0.025	0.006	< 0.001	0.002	0.000	0.052

## Abbreviations

PE: preeclampsia
EHR: electronic health record
UM: University of Michigan
IRB: Institutional Review Board
ICD: International Classification of Diseases
OR: odds ratio
CI: confidence interval
VIF: Variance Inflation Factor
Cox-PH: Cox Proportional-Hazard
HR: Hazard ratio
